# Supplementary material for: Drug repurposing screen identifies lonafarnib as respiratory syncytial virus fusion protein inhibitor
Source: Nat Commun. 2024 Feb 8;15:1173. doi: 10.1038/s41467-024-45241-y (PMC10853176; doi:10.1038/s41467-024-45241-y)
Supplement: Supplementary file 1 — Supplementary Information [file 41467_2024_45241_MOESM1_ESM.pdf]

Drug repurposing screen identifies lonafarnib as respiratory syncytial virus fusion protein inhibitor

Svenja M. Sake[1], Xiaoyu Zhang[1], Manoj Kumar Rajak[2,3], Melanie Urbanek-Quaing[1], Arnaud Carpentier[1], Antonia P. Gunesch[1], Christina Grethe[1], Alina Matthaei[1], Jessica Rückert[2], Marie Galloux[4], Thibaut Larcher[5], Ronan Le Goffic[4], Fortune Hontonnou[4], Arnab K. Chatterjee[6], Kristen Johnson[6], Kaycie Morwood[6], Katharina Rox[7,8], Walid A. M. Elgaher[9,10,11], Jiabin Huang[12], Martin Wetzke[13,14], Gesine Hansen[11,13,14], Nicole Fischer[12], Jean-Francois Eléouët[4], Marie-Anne Rameix-Welti[15], Anna K. H. Hirsch[9,10,11,16], Elisabeth Herold[3], Martin Empting[8,9,10,11], Chris Lauber[1,11], Thomas F. Schulz[2,8,11], Thomas Krey[2, 3,17,18], Sibylle Haid[1*] and Thomas Pietschmann[1,8,11,16*]

[1]*Institute for Experimental Virology, TWINCORE, Centre for Experimental and Clinical Infection Research, Hannover, Germany*

[2]*Institute of Virology, Hannover Medical School, Hannover, Germany*

[3]*Center of Structural and Cell Biology in Medicine, Institute of Biochemistry, University of Luebeck, Germany*

[4]*Université Paris-Saclay, INRAE, UVSQ, VIM, Jouy-en-Josas, France*

[5]*INRAE Oniris, PAnTher, APEX, Oniris, Nantes, France*

[6]*Calibr, Scripps Research, La Jolla, California, USA*

[7]*Department of Chemical Biology, Helmholtz Center of Infection Research, Braunschweig, Germany*

[8]*German Centre for Infection Research, Partner site Braunschweig-Hannover, Germany*

[9]*Helmholtz Institute for Pharmaceutical Research Saarland (HIPS) - HZI, Saarbrücken, Germany*

[10]*Department of Pharmacy, Saarland University, Saarbrücken, Germany*

[11]*Cluster of Excellence RESIST (EXC 2155), Hannover Medical School, Hannover, Germany*

[12]*Insitute for Medical Microbiology, Virology and Hygiene, University Medical Center Hamburg-Eppendorf, Hamburg, Germany*

[13]*Department for Pediatric Pneumology, Allergology and Neonatology, Hannover Medical School, Hannover, Germany*

[14]*German Center for Lung Research, Partner Site Hannover, BREATH, Germany*

[15]*Université Paris-Saclay, Université de Versailles St. Quentin; UMR 1173 (2I), INSERM; Assistance Publique des Hôpitaux de Paris, Hôpital Ambroise Paré, Laboratoire de Microbiologie, DMU15; Versailles, France*

[16]*Helmholtz International Lab for Anti-infectives, HZI, Braunschweig, Germany*

[17]*Centre for Structural Systems Biology (CSSB), Hamburg, Germany*

[18]*German Center for Infection Research, Partner Site Hamburg-Luebeck-Borstel-Riems, Germany*

Co-Corresponding authors

Sibylle Haid, Institute for Experimental Virology, TWINCORE Center of Experimental and Clinical Infection Research, Hannover, Germany; sibylle.haid@twincore.de

Thomas Pietschmann, Institute for Experimental Virology, TWINCORE Center of Experimental and Clinical Infection Research, Hannover, Germany; thomas.pietschmann@twincore.de

Supplementary Information

1. **Supplementary Figure S1.** Dose-response analysis of ReFRAME library hits.
2. **Supplementary Figure S2.** Molecular target categories and development stage of 21 primary hit compounds.
3. **Supplementary Figure S3.** Phylogenetic analysis of early passage RSV isolates from patients.
4. **Supplementary Figure S4.** Pharmacokinetic profile of lonafarnib after oral administration.
5. **Supplementary Figure S5.** Anomalous difference map identifies lonafarnib binding site.
6. **Supplementary Figure S6.** FACS gating strategy.
7. **Supplementary Table S1**. Data collection parameters for co-crystallography.
8. **Supplementary materials and methods**

9. **References of supplementary material**

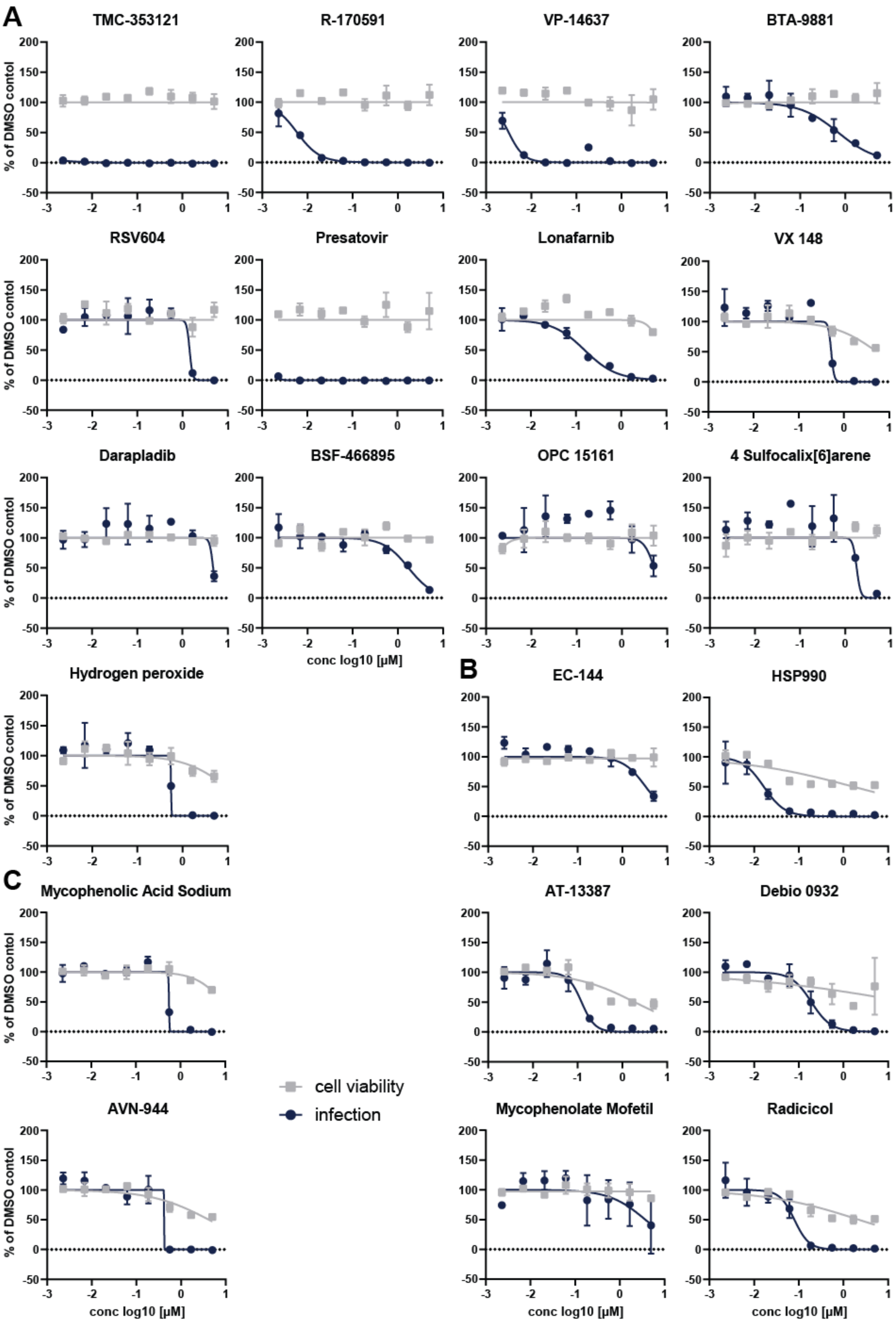
A
TMC-353121
R-170591
VP-14637
BTA-9881
RSV604
Presatovir
Lonafarnib
VX 148
Darapladib
BSF-466895
OPC 15161
4 Sulfocalix[6]arene
Hydrogen peroxide
B
EC-144
HSP990
AT-13387
Debio 0932
Mycophenolate Mofetil
Radicicol
C
Mycophenolic Acid Sodium
AVN-944
% of DMSO contol
conc log10 [μM]
cell viability
infection

Supplementary Figure S1: Dose-response analysis of ReFRAME library hits. HEp-2 cells were infected with a recombinant RSV subtype A GFP reporter virus in presence of indicated compound concentrations. 48 hours after inoculation GFP signal as a measure of infection (dark blue) was quantified and cell viability (grey) was determined using an MTT assay. Both were normalized to a DMSO control. **(A)** Compounds fulfilled previous hit criteria. **(B)** Compounds included due to floating MTT threshold **(C)** GFP signal in screening ≤ 0 % of DMSO control. Mean and standard deviation from one experiment (n=1) performed in duplicates are shown. Besides different RSV fusion inhibitors (TMC-353121, R-170591, VP-14637, BTA-9881, presatovir), the N-protein inhibitor RSV-604[1] was among the strongest RSV targeting antivirals in the dose-response analysis. We also found five molecules (EC-144, HSP990, AT-13387, Debio 0932 and radicicol) that were reported to inhibit heat shock protein 90 (HSP90). Finally, we identified darapladib, a phospholipase inhibitor and lonafarnib, an inhibitor of farnesyl-s-transferases. Source data are provided as a Source Data file.

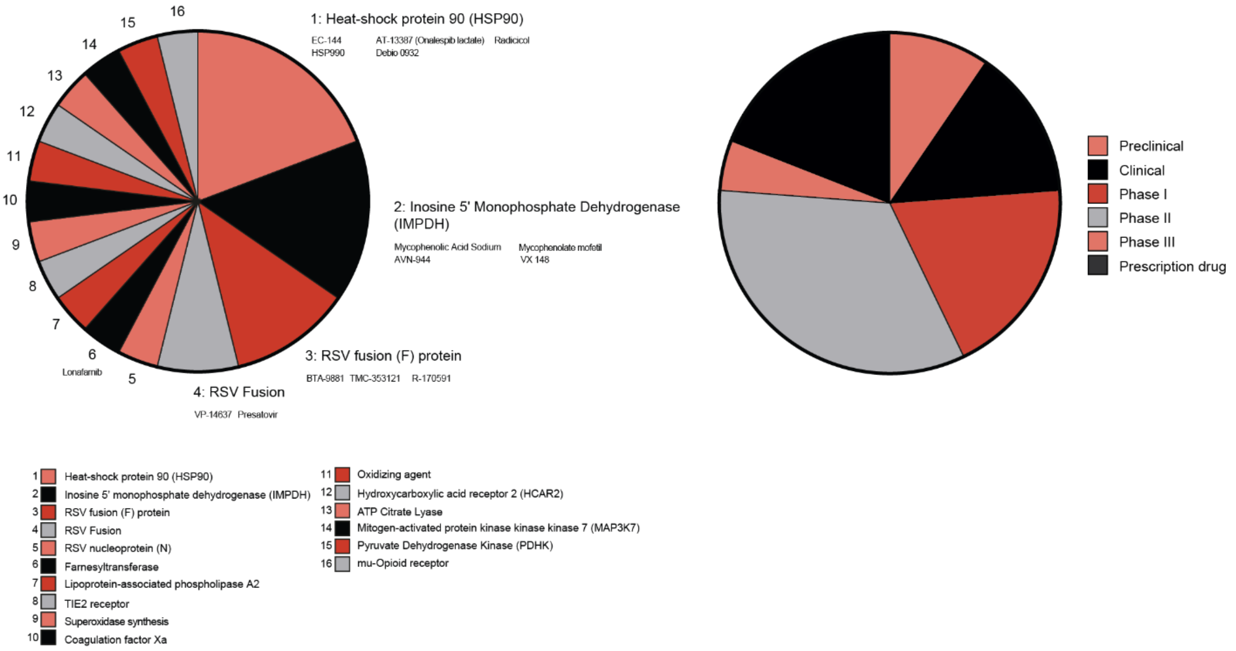

Supplementary Figure S2: Molecular target categories and development stage of 21 primary hit compounds. Metadata of the ReFRAME databases was used to determine the putative molecular targets and the stage of development of 21 hit compounds. In total, the 21 hits mapped to 16 distinct categories. The largest number of inhibitor candidates mapped to the HSP90 and IMPDH target categories.

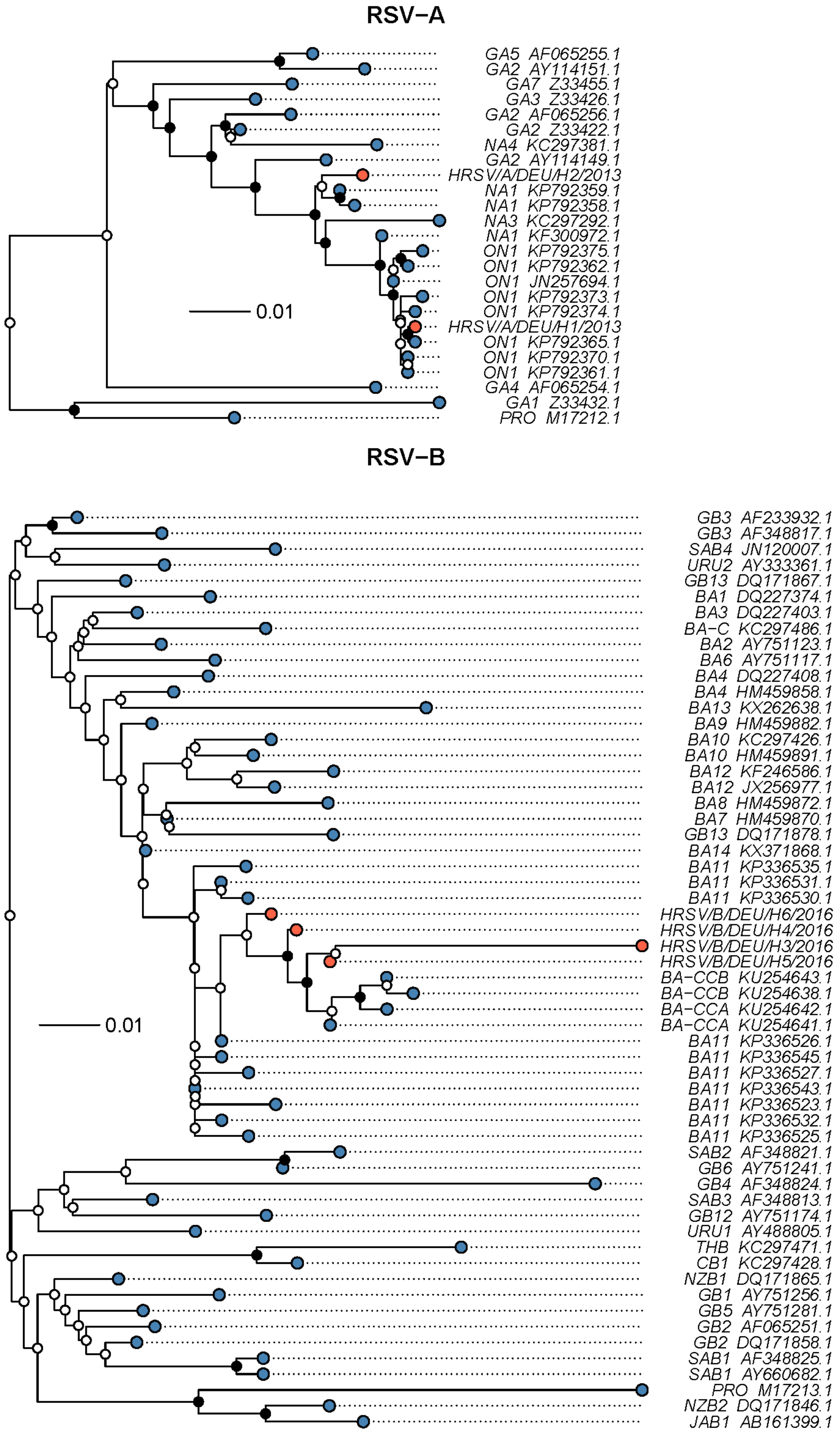
RSV−A
GA5 AF065255.1
GA2 AY114151.1
GA7 Z33455.1
GA3 Z33426.1
GA2 AF065256.1
GA2 Z33422.1
NA4 KC297381.1
GA2 AY114149.1
HRSV/A/DEU/H2/2013
NA1 KP792359.1
NA1 KP792358.1
NA3 KC297292.1
NA1 KF300972.1
ON1 KP792375.1
ON1 KP792362.1
ON1 JN257694.1
ON1 KP792373.1
ON1 KP792374.1
HRSV/A/DEU/H1/2013
ON1 KP792365.1
ON1 KP792370.1
ON1 KP792361.1
GA4 AF065254.1
GA1 Z33432.1
PRO M17212.1
0.01
RSV−B
GB3 AF233932.1
GB3 AF348817.1
SAB4 JN120007.1
URU2 AY333361.1
GB13 DQ171867.1
BA1 DQ227374.1
BA3 DQ227403.1
BA−C KC297486.1
BA2 AY751123.1
BA6 AY751117.1
BA4 DQ227408.1
BA4 HM459858.1
BA13 KX262638.1
BA9 HM459882.1
BA10 KC297426.1
BA10 HM459891.1
BA12 KF246586.1
BA12 JX256977.1
BA8 HM459872.1
BA7 HM459870.1
GB13 DQ171878.1
BA14 KX371868.1
BA11 KP336535.1
BA11 KP336531.1
BA11 KP336530.1
HRSV/B/DEU/H6/2016
HRSV/B/DEU/H4/2016
HRSV/B/DEU/H3/2016
HRSV/B/DEU/H5/2016
BA−CCB KU254643.1
BA−CCB KU254638.1
BA−CCA KU254642.1
BA−CCA KU254641.1
BA11 KP336526.1
BA11 KP336545.1
BA11 KP336527.1
BA11 KP336543.1
BA11 KP336523.1
BA11 KP336532.1
BA11 KP336525.1
SAB2 AF348821.1
GB6 AY751241.1
GB4 AF348824.1
SAB3 AF348813.1
GB12 AY751174.1
URU1 AY488805.1
THB KC297471.1
CB1 KC297428.1
NZB1 DQ171865.1
GB1 AY751256.1
GB5 AY751281.1
GB2 AF065251.1
GB2 DQ171858.1
SAB1 AF348825.1
SAB1 AY660682.1
PRO M17213.1
NZB2 DQ171846.1
JAB1 AB161399.1
0.01

Supplementary Figure S3. Phylogenetic analysis of early passage RSV isolates from patients. G gene sequence-based phylogenetic trees generated with the BioNJ method for RSV-A (top) and RSV-B (bottom). Genotypes according to Munoz-Escalante et al.[2, 3] and GenBank accession numbers are shown for RSV reference sequences, which are indicated by blue dots. Red dots highlight sequences generated in this study. Black dots at internal nodes indicate bootstrap support values of at least 70%; otherwise white dots are shown. The scale bars are in units of substitutions per site.

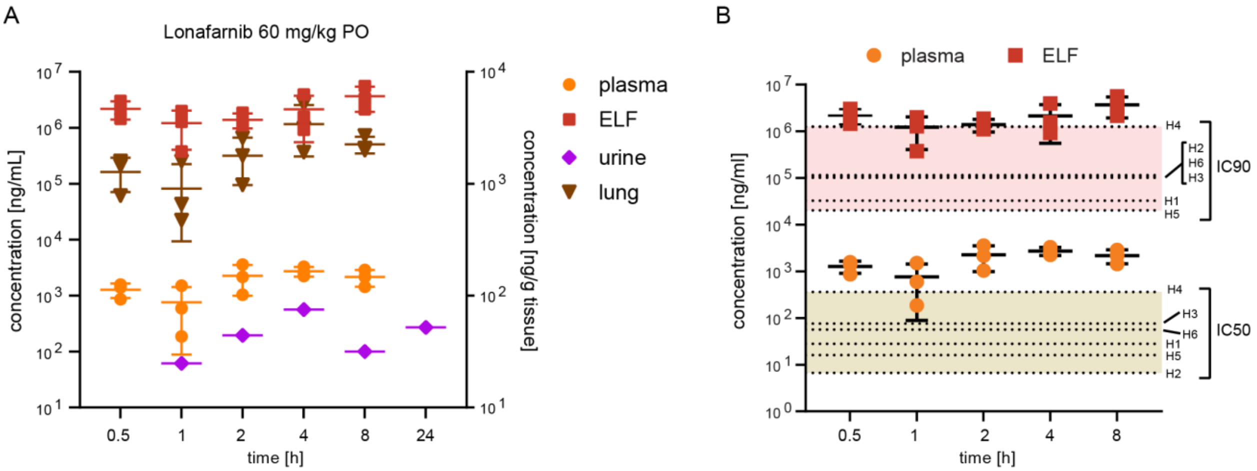

Supplementary Figure S4. Pharmacokinetic profile of lonafarnib after oral administration. Lonafarnib was administered via the oral route at 60 mg/kg. Groups of three mice were analyzed at each time point and means +/- SD and individual animals are plotted. N=1 for urine. (A) Compound levels for plasma, urine, ELF (all ng/ml) and lung tissue (ng/g) are displayed. (B) Total plasma levels as well as ELF levels are plotted in addition to the IC_{50} (beige) and IC_{90} values (reddish) of the RSV isolates. H1: HRSV/A/DEU/H1/2013; H2: HRSV/A/DEU/H2/2013; H3: HRSV/B/DEU/H3/2016; H4: HRSV/B/DEU/H4/2016; H5: HRSV/B/DEU/H5/2016; H6: HRSV/B/DEU/H6/2016. Source data are provided as a Source Data file.

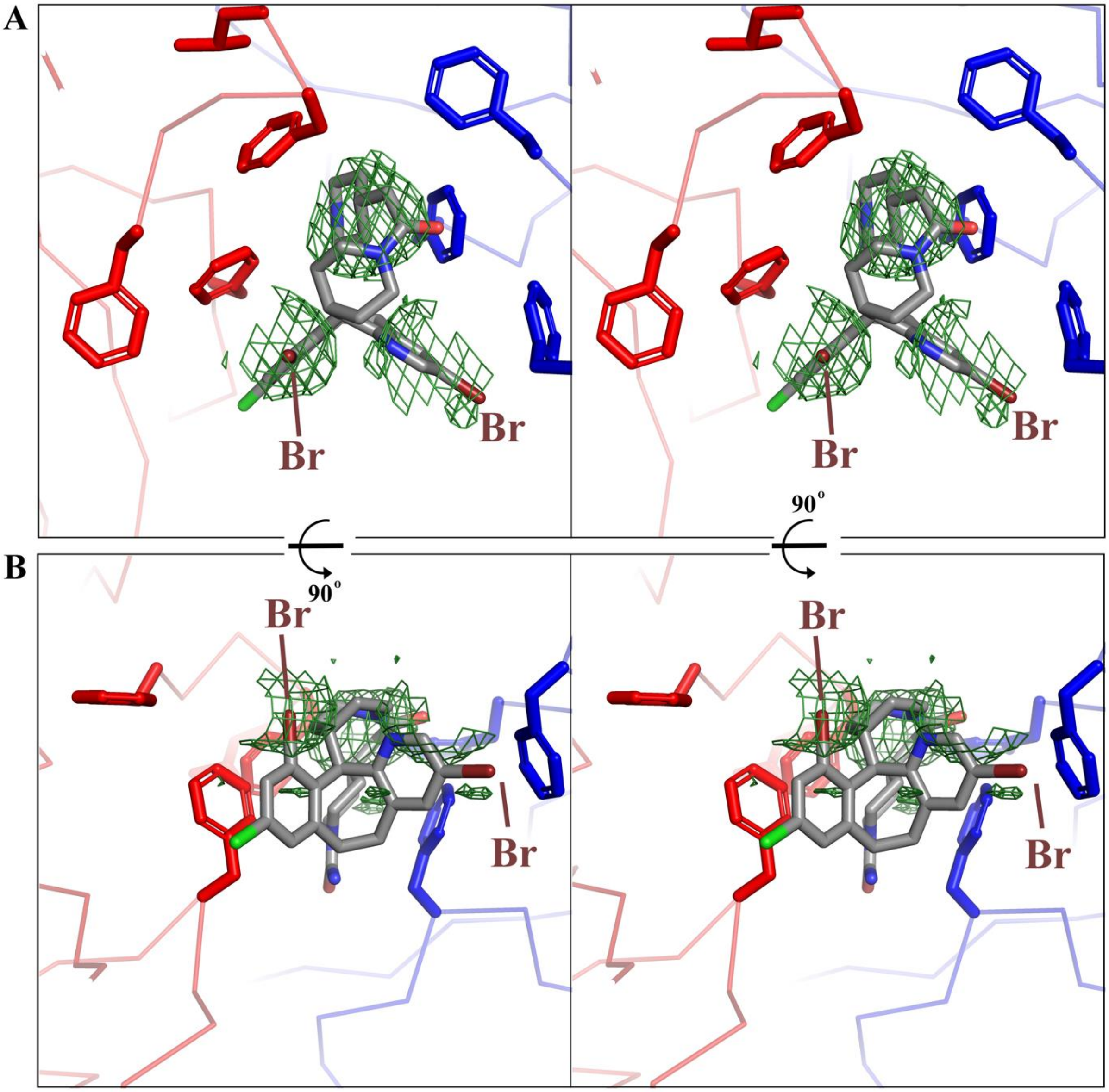

Supplementary Figure S5. Anomalous difference map identifies lonafarnib binding site. Stereo images of the top (A) and side views (B) of lonafarnib bound to the RSV F trimer including an anomalous difference map (green) contoured at 2.5 sigma around the ligand, indicating the positions of the bromine atoms within lonafarnib. One protomer is removed for clarity.

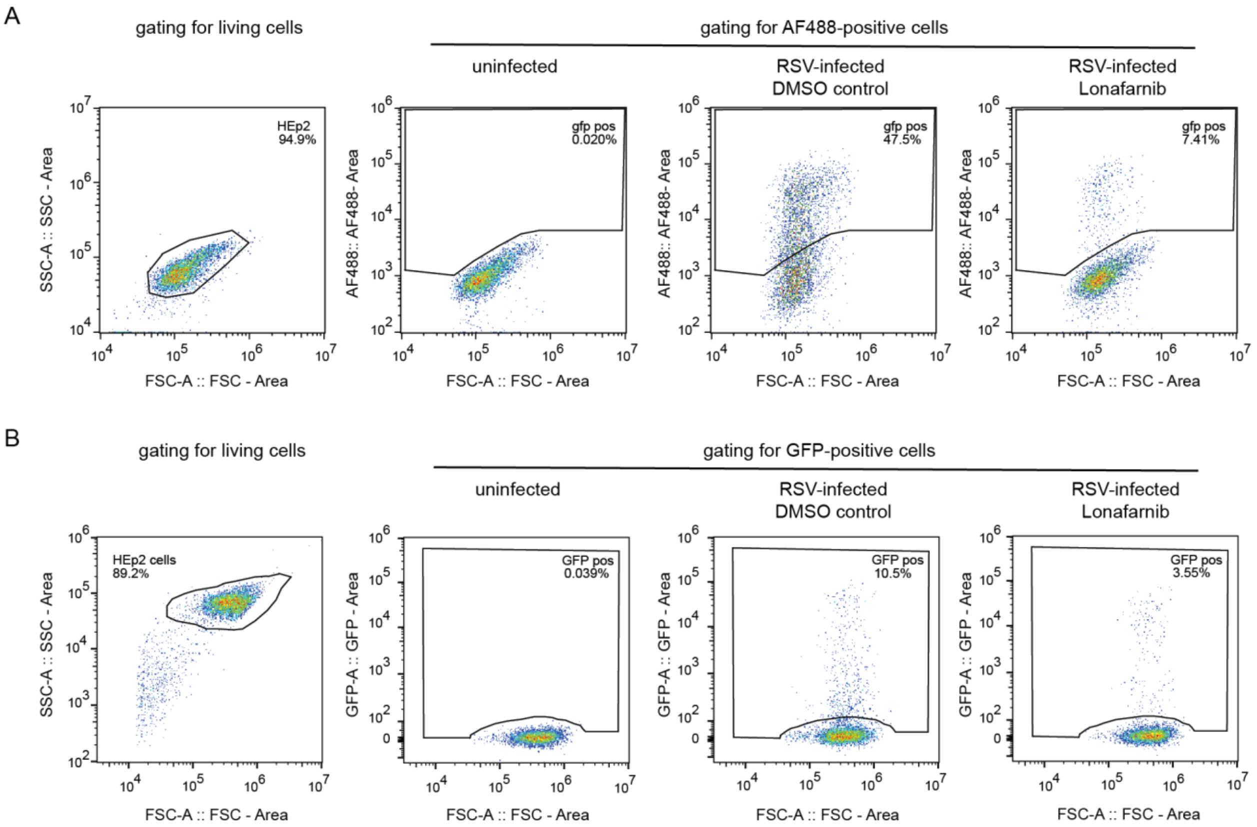

Supplementary Figure S6. Flow cytometry gating strategy. In a first step, living cells were gated according to their FSC-SSC profile. This population was then used for gating of (A) RSV-P/AlexaFluor488 positive cells or in case of infection with an RSV reporter virus (B) GFP-expressing, RSV-infected cells using an uninfected sample as negative control.

Table S1. Data collection and refinement statistics

	RSV F + Lonafarnib
**Data collection**[a]	
Space group	*H*3
Cell dimensions	
a, *b*, *c* (Å)	88.64 88.64 194.98
α, β, γ (°)	90.00 90.00 120.00
Resolution (Å)	50 – 2.29 (2.35 – 2.29)[b]
R_{merge}	0.238 (1.708)
I / σI	10.54 (1.66)
Completeness (%)	99.9 (99.0)
Redundancy	26.1 (19.0)
**Refinement**	
Resolution (Å)	26.21 – 2.289 (2.31 – 2.289)
No. reflections	25724
R_{work} / R_{free}	0.1984 / 0.2285
No. atoms	
Protein	3425
Ligand/ion	58
Water	177
B-factors	
Protein	50.3
Ligand/ion	100.13
R.m.s. deviations	
Bond lengths (Å)	0.007
Bond angles (°)	0.98

[a]Data are from one crystal.
[b]Values in parentheses are for highest-resolution shell.

7. Supplementary materials and methods

Dose-response analysis of primary hits

HEp-2 cells were seeded at a density of 1.5×10^4 cells/well in 96-well plates one day prior infection. Cells were infected with an MOI of 0.1 in the presence of indicated drug concentrations. 24h post inoculation, the supernatant containing the newly produced progeny viruses was used to inoculate HEp-2 cells seeded the day before (2nd round of infection) and the cells from the first round of infection were lysed (1% Triton X-100, 25 mM Gly-Gly, 15 mM $MgSO_4$, 4 mM EGTA, 1 mM DTT in H_2O). 72h post inoculation of cells with transferred supernatant from round one, the cells from the second round of infection were lysed. Luciferase activity was measured using a Berthold Centro plate reader LB 960 and MikroWin software (version 4.41). IC_{50}, IC_{90} and CC_{50} values were calculated with GraphPad Prism V7 and V8 (GraphPad software, San Diego, CA, USA). Therefore, the relative light unit (RLU) values were normalized to the positive and negative control using Excel software.

Cell-viability determination via MTT assay

Cells were seeded at 1.5×10^4 cells/well in a 96-well plate, treated with media containing the indicated concentrations of drugs and incubated at 37°C. At the time of viability measurement, supernatant was replaced by 50 µl prewarmed media containing 1 mg/ml MTT (3-(4,5-dimethylthiazol-2-yl)-2,5-diphenyl tetrazolium bromide), incubated for 30-60 min at 37°C prior to cell lysis by addition of 50 µl isopropanol per well. Absorbance at 570 nm and 630 nm was measured using a BioTek Synergy Microplate reader.

Intracellular immunofluorescence staining and flow cytometry

Treated cells were trypsinized and transferred to a V-shaped 96-well plate. Cells were pelleted at 300 × g for 5 min prior to fixation in 0.5% paraformaldehyde/1%FCS/PBS and permeabilization in 0.1% saponin /PBS for 20 min on ice. Cells were again pelleted and supernatant was discarded, then cells were resuspended in a phosphoprotein targeting antibody (murine antibody RSV-P#26 D2G6C5; final concentration 2 µg/ml) and incubated for 60 min. After washing the cells twice with PBS an Alexa-Fluor488 coupled anti-mouse antibody (Invitrogen; #A32723; final concentration 0.01 mg/ml in permeabilization buffer) was added to the cells for another 30 min. Unbound secondary antibody was removed by PBS washes and cells were analyzed on a Accuri C6 cytometer (BD, Heidelberg, Germany) or a SA3800 spectral analyzer (SONY) and results were analyzed using FlowJo V10 (Tree Star, Ashland, USA).

Pseudoparticle assay

Lentiviral pseudotyped viral particles were produced as described before [4]. Briefly, producer cells HEK293T were seeded in poly-L-lysine coated 10 cm-dishes at 3×10^6 cells/plate in DMEM complete medium. The next day, cells were co-transfected with plasmids encoding the HIV-based packaging genes gag-pol (pRV 8.74) [5], the transfer plasmid encoding the firefly reporter gene, as well as an envelope plasmid encoding the wildtype glycoprotein of RSV (RSV F protein), resistance-associated mutations of RSV F, VSV-G or the empty vector pcDNA3.1. A total of 15 µg DNA were co-transfected using polyethyleneimine (PEI). After 21h post transfection, sodium-butyrate was added and medium was changed after another two hours incubation at 37°C. After 30h post transfection, pseudoparticle-containing supernatant was harvested and filtered (0.45 µm pore size). Target cells Huh-7.5 were seeded in 12-well plates at a density of 8×10^4 cells/well in 1 ml DMEM complete the day before transduction and incubated at 37°C. Cells were transduced with the respective pseudotyped particles and lonafarnib in indicated concentrations or DMSO and

incubated over night at 37°C. The next day, medium was exchanged and cells were lysed after another 48h incubation followed by firefly luciferase activity measurement.

Plaque assay

HEp-2 cells were seeded at a density of 1 × 10^5 cells/well of a 12-well plate the day prior to inoculation. Cells were inoculated with a clinical RSV-A strain (HRSV/A/DEU/H1/2013 [6]) in the presence of indicated drug concentrations for 2h at 37°C. After 2h inoculation, inoculum was removed and replaced by 3 ml of 0.6% Avicel (FMC Biopolymer)/2% serum/MEM containing the indicated concentrations of the drug. Cells were incubated for 6 days at 37°C followed by crystal violet staining (10% crystal violet oxalate solution (reference number HT90132, Sigma-Aldrich Co., St. Louis, MO, USA), 3.7% PFA, 20% Ethanol in H_2O) of the cell layer and determination of plaque size and number.

hCoV-229E infection

Huh-7.5_Fluc cells were seeded at a density of 2 × 10^4 cells/well one day prior to infection. The next day cells were inoculated with hCoV-229E-Rluc reporter virus in the presence of indicated concentrations of compounds or DMSO as solvent control and incubated at 33°C for 48h. At cell lysis, media was removed, cells washed twice in PBS and subsequently lysed in 0.5% TritonX-100/PBS. Virus replication was analyzed via measurement of the Renilla-luciferase reporter activity whereas cell viability was analyzed by quantification of the Firefly-luciferase activity using a plate luminometer (Berthold LB960).

HDV production assay

Infectious HDV were produced as previously described [7]. Briefly, Huh-7 cells overexpressing the HDV entry receptor NTCP (Huh7-hNTCP) were transfected with both plasmids pSVLD3 (containing a trimer of the HDV genome, genotype 1) [8] and pT7HB2.7 (encoding the HBV surface antigen, genotype D) [9] using the FuGENE transfection reagent (3.5:1 FuGENE to DNA ratio). Transfected cells were then kept 10 days in culture, treated or not with 0.1 or 1 μM of lonafarnib and tipifarnib throughout the experiment. Expression of the HDV antigen (HDAg) was confirmed at day 10 post transfection by IFA, using the antibody HDAg#280 (SySy, Göttingen, Germany) diluted 1 in 1000, as described in [10]. At day 10 post transfection, supernatants were collected, filtered and their infectious titer investigated. To do so, we incubated naive Huh7-hNTCP with serial dilution of transfected cells treated or not with antiviral drugs for 5 days, followed by staining for HDAg and quantification of positive foci using an Olympus IX81 microscope.

Time-of-addition assay

HEp-2 cells were seeded in 96-well-plates at a density of 1.5×10^4 per well one day prior to treatment. The following day, cells were incubated with HRSV/A/DEU/H1/2013 (MOI of 1) for 2 hours at 37°C, with compounds added at different time points throughout infection. Pre-incubation: Compounds were added 4 hours prior to virus infection, and were washed away with PBS before virus incubation. Co-incubation: Compounds were added in presence of virus for 2 hours, before washing away with PBS and replenishing with fresh media. Post-incubation I: After aspirating the virus containing media after 2 hours, cells were washed once with PBS and replenished with compound containing media. Post-incubation II: After aspirating the virus containing media after 2 hours, cells were washed once with PBS and replenished with fresh media, then after 2 hours, media was aspirated and replaced with compound containing media. Post-incubation III: After aspirating the virus containing media after 2 hours, cells were washed

once with PBS and replenished with fresh media, then after 4 hours, media was aspirated and replaced with compound containing media. 24 hours post virus infection, cells were detached and fixed for intracellular staining of RSV-P (murine antibody RSV-P#26 D2G6C5; final concentration 2 µg/ml) followed by staining with AlexaFluor488 anti-mouse secondary antibody (Invitrogen; #A32723; final concentration 0.01 mg/ml) and subsequent flow cytometry.

RSV replicon assay

BSR-T7/5 cells stably expressing the RNA polymerase of bacteriophage T7 [11] were seeded in 6-well-plates at a density of 5×10^5 cells per well. One day later, plasmids encoding for RSV phosphoprotein P, nucleoprotein N, polymerase L and anti-transcription-termination factor M2-1 were mixed with a plasmid encoding for a sub-genomic replicon of RSV strain A2 with a firefly luciferase reporter. These plasmids all contain a T7 promotor upstream of gene coding region. Solutions containing all plasmids but the L protein encoding plasmid or containing only one plasmid encoding for a T7 polymerase independent firefly luciferase (pWPI-Fluc), were prepared as negative and positive controls, respectively. Transfection was performed using Lipofectamine® 2000 reagent according to manufacturer’s instructions. Four hours after transfection, cells were reseeded in 96-well-plates at a density of 1×10^4 cells per well. Compounds dissolved in medium were added to the cells. After 72 hours incubation at 37°C, cells were lysed for subsequent luminescence quantification.

Therapeutic treatment of virus infected A549 cells

A549 cells were seeded in 96-well-plates at a density of 1×10^4 cells per well one day prior to infection. One day later, cells were infected with rHRSV-A-GFP at a MOI of 0.01. 24 hours post

infection, cells were washed with PBS and replenished with compounds containing media. Cells were harvested at 48h, 72h, 96h, and 120h post infection and fixed for subsequent flow cytometry.

Virus adaptation

HEp-2 cells were inoculated with a recombinant RSV-A-GFP reporter virus at an MOI of 0.5 in the presence of 0.5, 1, 2 or 4 µM of the compound respectively or 1% DMSO as solvent control. Three hours after inoculation the inoculum was replaced by fresh medium containing the same drug concentration as during viral infection and the cells were cultured at 37°C. Depending on syncytia formation after 48h, one compound concentration was chosen for further passaging. Therefore, the supernatant of the respective well was harvested, mixed in a 5:1 (volume/volume) ratio with a stabilizing solution (500mM $MgSO_4$, 250mM HEPES, pH 7.5), and cleared from cell debris by centrifugation at 1000 × g for 5 min. Half of the supernatant was snap-frozen in liquid nitrogen for further analysis and the other half was used to inoculate naïve HEp-2 cells in the presence of the same compound concentration as before. After 3 hours inoculum was removed and replaced by fresh media with increased concentrations of the same compound as initially. Cells were incubated for another 48 to 96h depending on syncytia formation and further passaging. After 10 rounds of passaging with constantly increased drug concentrations the newly generated virus population was analyzed by next generation sequencing.

Next generation sequencing

Sequencing analysis of resistance mutations were conducted as described recently [12]. Briefly, ribosomal RNA was depleted from samples using the NEBNext rRNA Depletion Kit v2 (New

England Biolabs). Sequencing libraries were prepared with the NEB Ultra II RNA library preparation (New England Biolabs) according to manufacturer´s instructions. Each library was multiplex-sequenced on an Illumina MiSeq instrument (300 cycles, PE protocol) with ca. 2,000,000 reads per sample. Adapter sequences of the reads and bases with a score of less than Q30 were trimmed. Reads shorter than 36 nt were removed using Trimmomatic v0.36 [13]. Paired-end reads were identified as mouse sequences by mapping to the host genome (mm10, GCA_000001635.2) using the Bowtie2 package [14] and subsequently removed from the analysis. Unmapped reads were then aligned to the sequence of the recombinant HRSV subtype A GFP reporter virus (GenBank accession number MK816924) using Novoalign V3.07.00 (http://www.novocraft.com). The tool MarkDuplicate available in the Picard package (http://broadinstitute.github.io/picard/) was used to remove duplicate sequences from the alignment. The derived alignment was fed into V-Phaser2 [15] for intra-individual single nucleotide variation (iSNV) identification. In the variant identification, we only considered variants supported by at least five reads on each strand, and the ratio of the number of reads on the two strands is smaller than 10. The accession numbers for the sequencing raw reads in the NCBI Sequence Read Archive (SRA) are SRR22746624, SRR22746625, and SRR22746626 (https://www.ncbi.nlm.nih.gov/sra).

Nanopore sequencing of RSV clinical isolates

Genomic sequences of RSV clinical isolates used in this study were determined by nanopore sequencing. Briefly, viral RNAs were extracted from virus containing supernatants using High Pure Viral RNA Kit (Roche, Cat. Num.: 11858882001). Library preparation was performed using ONT PCR-cDNA barcoding kit (SQK-PCB109) according to manufacturer's instructions. The library was sequenced on a R9.4.1 flow cell. The sequencing raw data (.fast5) was basecalled by

Guppy in HAC mode. The sequencing data was then adapter trimmed and demultiplexed for each virus sample. Reads were aligned to either RSV A or RSV B reference genomes (KU950592, MW582529) by EPI2ME Labs workflow wf-alignment (https://github.com/epi2me-labs/wf-alignment). The aligned reads were visualized in iGV and consensus sequences generated. The data for this study have been deposited in the European Nucleotide Archive (ENA) at EMBL-EBI under accession number PRJEB63686.

RSV genotyping

We utilized the G gene sequence and genotype information from Munoz-Escalante et al. [2, 3] and analyzed RSV-A and RSV-B separately. We aligned the nucleotide reference sequences together with the sequences generated in this study using MAFFT v7.310 with parameter --maxiterate 1000 [16], followed by manual alignment curation. In case of identical sequences from the same genotype, we kept only one sequence. We reconstructed phylogenetic trees using the BioNJ method under the J-C model with 100 bootstraps [17]. We inferred genotypes of the RSV sequences generated in this study based on their clustering in the phylogeny and genetic distance to known genotypes.

Surface Plasmon Resonance

Recombinant RSV F protein derived from the A2 strain in a prefusion conformation stabilized by the structure-based design of disulfide (DS) and cavity-filling (Cav1) mutations [18] was produced in Drosophila melanogaster S2 cells. A gene encoding the stabilized glycoprotein was cloned into a modified Drosophila S2 expression vector described previously and transfection was performed as reported earlier [19]. For large-scale production, cells were induced with 4 mM $CdCl_2$ at a density of approximately 6×10^6 cells/ml for 6 days, pelleted, and the soluble trimeric F ectodomain was purified by affinity chromatography from the supernatant using a StrepTactin Superflow column

followed by size exclusion chromatography using a Superose 6 column equilibrated in 10 mM TRIS, 150 mM NaCl. Pure protein was concentrated to approximately 5 mg/ml. Binding affinity determinations were carried out using a Reichert SR7500DC surface plasmon resonance spectrometer (Reichert Technologies, Depew, NY, USA), and medium density carboxymethyl dextran hydrogel CMD500M sensor chips (XanTec Bioanalytics, Düsseldorf, Germany). Milli-Q water was used as the immobilization buffer. Tris-buffered saline with 0.05% Tween 20 (TBST) buffer (10 mM Tris, 150 mM NaCl, 0.05% v/v Tween 20, pH 8.0) containing 2% v/v DMSO was used as the running buffer for binding studies. All running buffers were filtered and degassed prior to use. The RSV pre-fusion F protein was immobilized in the first flow cell via amine-coupling [20], whereas the second flow cell was left blank as a reference. The system was initially primed with borate buffer 100 mM (pH 9.0), then the carboxymethyldextran matrix was activated by a 1:1 mixture of *N*-ethyl-*N'*-(3-dimethylaminopropyl)carbodiimide hydrochloride (EDC) 100 mM and *N*-hydroxysuccinimide (NHS) 100 mM at a flow rate of 10 μl/min for 7 min. RSV F protein was diluted to a final concentration of 50 μg/ml in 10 mM sodium acetate buffer (pH 4.5), and was injected at a flow rate of 5 μl/min for 10 min. The non-reacted surface was quenched by 1 M ethanolamine hydrochloride (pH 8.5) at a flow rate of 25 μl/min for 3 min. A series of 10 TBST buffer injections was run initially on both reference and active surfaces to equilibrate the system resulting in a stable immobilization level of approximately 6000 μ refractive index unit (μRIU). Binding experiments were performed at 20°C. Stock solutions of the compounds in DMSO were diluted with TBST buffer to attain seven concentrations (1.56–100 μM, final DMSO concentration of 2% v/v) and were injected at a flow rate of 40 μl/min. Single cycle kinetics were applied for K_D determinations. The association time was set to 60 s, and the dissociation phase was recorded for 120 s. Differences in the bulk refractive index due to DMSO were corrected by a calibration curve

(seven concentrations: 1.2–3% v/v DMSO in the running buffer). Data processing and analysis were performed by Scrubber software (Version 2.0c, 2008, BioLogic Software). Sensorgrams were calculated by sequential subtractions of the corresponding curves obtained from the reference flow cell and the running buffer (blank). SPR responses are expressed in the resonance unit (RU). The equilibrium dissociation constant (K_D) of lonafarnib was calculated by global fitting of the association and dissociation curves. Data were obtained from two independent experiments.

$TCID_{50}$

1.5×10^4 HEp-2 cells/well were seeded into a 96-well plate (200 µl/well). 24 hours post seeding, serial dilutions of a virus stock (six replicates per dilution) were used to inoculate the cells. Pipet tips were changed for every dilution to minimize carryover. 24 hours post inoculation, cells were fixed in ice-cold methanol for immunohistochemical staining of the HRSV phosphoprotein using a mouse hybridoma supernatant (#26D6G5C6) and anti-mouse HRP-conjugated secondary antibodies (Sigma-Aldrich; #A4416). HRSV P positive cells were detected with a microscope and virus titer was calculated as tissue culture infection dose 50% ($TCID_{50}$) per milliliter by the method from Spearman and Karber [21].

Immunofluorescence analysis of HEp-2 cells infected with clinical RSV isolates

HEp-2 cells were seeded on glass cover slips (4×10^4 cells/well) one day prior to infection with clinical RSV isolates at an MOI of 0.1. Viral inoculum was removed 4h after start of inoculation and cells were treated with indicated concentrations of drugs and incubated at 37°C for 48h. Cells were fixed in 3% paraformaldehyde/PBS followed by immunostaining using an anti-RSV-P

antibody (murine antibody RSV-P#26 D2G6C5; final concentration 1 µg/ml), AlexaFluor488 anti-mouse secondary antibody (Invitrogen; #A32723; final concentration 2 µg/ml) and DAPI nuclear stain. Pictures were taken at the FV3000 microscope using a 10-fold magnification.

Analysis of RSV-F protein induced syncytia area

293T cells were seeded on PLL-coated 96-well plates (1×10^4 cells/well) one day prior to co-transfection of cells with 0.07 µg Venus-GFP and 0.01 µg RSV-F expressing plasmids per well using polyethyleneimine as transfection reagent. 6h post transfection, a media change containing the drugs was performed and the cells were incubated for 48h at 37°C. Four pictures per well were taken using an Incucyte SX5 (Sartorius; V2022B) at a 10-fold magnification. Syncytia area were analyzed using the Fiji software by setting equal threshold values to all pictures followed by automatic particles size determination. Syncytia smaller than 100 μm^2 were excluded from the analysis. For immunofluorescence analysis of RSV-F and Venus-GFP co-transfected 293T cells, cells were fixed in 3% paraformaldehyde 72h post transfection, permeabilized with 0.5% Triton X100/PBS and stained for RSV-F protein (Palivizumab; AbbVie; ChB 34180TXF; final concentration 10 µg/ml) and a secondary anti-human AlexaFluor647 conjugated antibody (life technologies; #A21445; final concentration 0.01 mg/ml) prior to unspecific labeling of the nuclei with Dapi. Cover slips were mounted using Fluoromount-G (Southern Biotech; #0100-01) and fluorescence imaging was performed on an Olympus FA3000 microscope at 100x magnification (oil).

Drug-drug interaction assay

1×10^4 HEp-2 cells were seeded in 96-well plates one day prior to infection with a recombinant RSV reporter virus in the presence of various concentrations of the one inhibitor alone or in combinations of both inhibitors. Cells were incubated for 24h at 37°C before cells were collected, lysed and luminescence was quantified. Baseline luminescence was measured in uninfected cells and was subtracted from all values and the measurements were normalized to solvent treated, infected controls. From individual compound treatments, a theoretical additive effect was calculated for all used concentrations as described by Prichard and Shipmen [22]. The calculated theoretical additive effect was subtracted from the measured combined effect of the two compounds.

Prophylactic treatment of virus infected ALI cultures

BCi-NS1.1 cells were seeded on transwells and differentiated into a pseudostratified respiratory epithelia as described elsewhere [23]. One hour prior to virus infection, cells were pretreated from the basolateral compartment with the indicated concentrations of compound. Cells were inoculated from apical with an RSV A GFP reporter virus in the presence of compound and incubated for 1h at 37°C. After 1h, virus inoculum was removed and cells were washed twice with HBSS to remove unbound virus. Cells were incubated at 37°C and treated twice daily from the apical side for 1h (8h in between apical treatment). Media from the basolateral compartment containing the compounds constantly was replaced on a daily basis. Once daily, prior to apical treatment, 200 µl HBSS was added to the apical cell layer, incubated for 30 min at 37°C and harvested for quantification of newly produced progeny virus.

Therapeutic treatment of virus infected ALI cultures

BciNS1.1 cells were seeded on transwells and differentiated into a pseudostratified respiratory epithelia as described elsewhere [23]. Prior to infection, the apical surface of the cells was washed with 200 µl HBSS to remove mucus. Cells were inoculated from apical with a clinical RSV-A isolate (HRSV/A/DEU/H1/2013; MOI 0.1) for 2h at 37°C. After 2h, virus inoculum was removed and the cells were washed twice with HBSS and further incubated at 37°C. Starting 24h post inoculation, cells were treated from basolateral with indicated concentrations of compounds on a daily basis. Once daily, the newly produced progeny virus was collected by apical washes with HBSS and a LDH toxicity assay (Sigma; TOX7) was performed from the basolateral media according to the instructions of the manufacturer. Viral RNA from apical washes was purified using the Maxwell Viral Nucleic acid kit (Promega) and viral genome equivalents were quantified by primer/probe specific qRT-PCR analysis (Roche, LightCycler480).

RNA extraction and qRT-PCR

Total RNA from supernatant was purified using the High Pure Viral RNA kit (Roche) and from cell lysates using the NucleoSpin RNA kit (Macherey-Nagel) according to the manufacturers. Purified RNA was eluted in 50 µl or 40 µl elution buffer respectively and subsequently used for qRT-PCR analysis using a Roche LightCycler 480 machine. Therefore, 5 µl of purified RNA was mixed with the LightCycler 480 RNA Master hydrolysis probes kit (Roche) and RSV-specific or GAPDH specific primer and probe combinations. RNA was reverse transcribed into DNA and further amplified in a one-step reaction (HRSV-A-fwd 5´-AgATCAACTTCTgTCATCCAgCAA; HRSV-A-rev 5´-TTCTgCACATCATAATTAggAg; HRSV-A probe 5´-6FAM-CACCATCCAACggAgCACAggAgAT-BBQ; GAPDH-fwd 5'-gAAggTgAAggTCggAgTC ; GAPDH-rev 5´- gAAgATggTgATgggATTTC; GAPDH probe 5’-LC640-CAAgCTTCCCgTTCTCAgCCT-BBQ (TibMolBiol, Berlin, Germany).

8. References of the supplementary material

1. Chapman J, *et al.* RSV604, a novel inhibitor of respiratory syncytial virus replication. *Antimicrob Agents Chemother* **51**, 3346-3353 (2007).

2. Munoz-Escalante JC, Comas-Garcia A, Bernal-Silva S, Noyola DE. Respiratory syncytial virus B sequence analysis reveals a novel early genotype. *Sci Rep* **11**, 3452 (2021).

3. Munoz-Escalante JC, Comas-Garcia A, Bernal-Silva S, Robles-Espinoza CD, Gomez-Leal G, Noyola DE. Respiratory syncytial virus A genotype classification based on systematic intergenotypic and intragenotypic sequence analysis. *Sci Rep* **9**, 20097 (2019).

4. Haid S, Grethe C, Bankwitz D, Grunwald T, Pietschmann T. Identification of a Human Respiratory Syncytial Virus Cell Entry Inhibitor by Using a Novel Lentiviral Pseudotype System. *J Virol* **90**, 3065-3073 (2015).

5. Dull T, *et al.* A third-generation lentivirus vector with a conditional packaging system. *J Virol* **72**, 8463-8471 (1998).

6. Blockus S, *et al.* Labyrinthopeptins as virolytic inhibitors of respiratory syncytial virus cell entry. *Antiviral Res* **177**, 104774 (2020).

7. Sureau C. The use of hepatocytes to investigate HDV infection: the HDV/HepaRG model. *Methods Mol Biol* **640**, 463-473 (2010).

8. Kuo MY, Chao M, Taylor J. Initiation of replication of the human hepatitis delta virus genome from cloned DNA: role of delta antigen. *J Virol* **63**, 1945-1950 (1989).

9. Sureau C, Fournier-Wirth C, Maurel P. Role of N glycosylation of hepatitis B virus envelope proteins in morphogenesis and infectivity of hepatitis delta virus. *J Virol* **77**, 5519-5523 (2003).

10. Buchmann B, *et al.* A screening assay for the identification of host cell requirements and antiviral targets for hepatitis D virus infection. *Antiviral Res* **141**, 116-123 (2017).

11. Buchholz UJ, Finke S, Conzelmann KK. Generation of bovine respiratory syncytial virus (BRSV) from cDNA: BRSV NS2 is not essential for virus replication in tissue culture, and the human RSV leader region acts as a functional BRSV genome promoter. *J Virol* **73**, 251-259 (1999).

12. Sake SM, *et al.* Respiratory Syncytial Virus Two-Step Infection Screen Reveals Inhibitors of Early and Late Life Cycle Stages. *Antimicrob Agents Chemother*, e0103222 (2022).

13. Bolger AM, Lohse M, Usadel B. Trimmomatic: a flexible trimmer for Illumina sequence data. *Bioinformatics* **30**, 2114-2120 (2014).

14. Langmead B, Salzberg SL. Fast gapped-read alignment with Bowtie 2. *Nat Methods* **9**, 357-359 (2012).

15. Yang X, Charlebois P, Macalalad A, Henn MR, Zody MC. V-Phaser 2: variant inference for viral populations. *BMC Genomics* **14**, 674 (2013).

16. Katoh K, Standley DM. MAFFT multiple sequence alignment software version 7: improvements in performance and usability. *Mol Biol Evol* **30**, 772-780 (2013).

17. Gascuel O. BIONJ: an improved version of the NJ algorithm based on a simple model of sequence data. *Mol Biol Evol* **14**, 685-695 (1997).

18. McLellan JS, *et al.* Structure-based design of a fusion glycoprotein vaccine for respiratory syncytial virus. *Science* **342**, 592-598 (2013).

19. Krey T, *et al.* The disulfide bonds in glycoprotein E2 of hepatitis C virus reveal the tertiary organization of the molecule. *PLoS Pathog* **6**, e1000762 (2010).

20. Hartman AM, Elgaher WAM, Hertrich N, Andrei SA, Ottmann C, Hirsch AKH. Discovery of Small-Molecule Stabilizers of 14-3-3 Protein-Protein Interactions via Dynamic Combinatorial Chemistry. *ACS Med Chem Lett* **11**, 1041-1046 (2020).

21. Vieyres G, Pietschmann T. Entry and replication of recombinant hepatitis C viruses in cell culture. *Methods* **59**, 233-248 (2013).

22. Prichard MN, Shipman C, Jr. A three-dimensional model to analyze drug-drug interactions. *Antiviral Res* **14**, 181-205 (1990).

23. Jonsdottir HR, Dijkman R. Characterization of human coronaviruses on well-differentiated human airway epithelial cell cultures. *Methods Mol Biol* **1282**, 73-87 (2015).
